# The Glymphatic Hypothesis of Glaucoma: A Unifying Concept Incorporating Vascular, Biomechanical, and Biochemical Aspects of the Disease

**DOI:** 10.1155/2017/5123148

**Published:** 2017-08-29

**Authors:** Peter Wostyn, Veva De Groot, Debby Van Dam, Kurt Audenaert, Hanspeter Esriel Killer, Peter Paul De Deyn

**Affiliations:** ^1^Department of Psychiatry, PC Sint-Amandus, Beernem, Belgium; ^2^Department of Ophthalmology, Antwerp University Hospital, Antwerp, Belgium; ^3^Laboratory of Neurochemistry and Behavior, Institute Born-Bunge, University of Antwerp, Department of Biomedical Sciences, Antwerp, Belgium; ^4^Department of Neurology and Alzheimer Research Center, University of Groningen and University Medical Center Groningen, Groningen, Netherlands; ^5^Department of Psychiatry, Ghent University Hospital, Ghent, Belgium; ^6^Department of Ophthalmology, Kantonsspital Aarau, Aarau, Switzerland; ^7^Department of Neurology and Memory Clinic, Middelheim General Hospital (ZNA), Antwerp, Belgium

## Abstract

The pathophysiology of primary open-angle glaucoma is still largely unknown, although a joint contribution of vascular, biomechanical, and biochemical factors is widely acknowledged. Since glaucoma is a leading cause of irreversible blindness worldwide, exploring its underlying pathophysiological mechanisms is extremely important and challenging. Evidence from recent studies appears supportive of the hypothesis that a “glymphatic system” exists in the eye and optic nerve, analogous to the described “glymphatic system” in the brain. As discussed in the present paper, elucidation of a glymphatic clearance pathway in the eye could provide a new unifying hypothesis of glaucoma that can incorporate many aspects of the vascular, biomechanical, and biochemical theories of the disease. It should be stressed, however, that the few research data currently available cannot be considered as proof of the existence of an “ocular glymphatic system” and that much more studies are needed to validate this possibility. Even though nothing conclusive can yet be said, the recent reports suggesting a paravascular transport system in the eye and optic nerve are encouraging and, if confirmed, may offer new perspectives for the development of novel diagnostic and therapeutic strategies for this devastating disorder.

## 1. Introduction

Glaucoma is one of the leading causes of irreversible blindness worldwide [1–3]. Primary open-angle glaucoma (POAG), the most common type, is characterized by the progressive degeneration of retinal ganglion cells (RGCs) and their axons in the optic nerve, resulting in structural changes in the optic nerve head and corresponding visual field defects [4]. The lamina cribrosa, a sieve-like structure in the posterior part of the sclera that allows passage of the RGC axons and central retinal vessels, seems to be the primary site of axonal injury in glaucoma [5]. Although the underlying pathophysiology of glaucomatous optic neuropathy (GON) remains elusive, elevated intraocular pressure (IOP) is considered the most important modifiable risk factor [6]. However, in a significant proportion of patients designated normal-tension glaucoma (NTG), the disease occurs in spite of normal IOP and thus other risk factors must also be involved in the optic neuropathy of POAG [6].

Although the mechanism(s) underlying optic nerve injury in glaucoma remain poorly understood, at least three theories have been suggested, including the vascular, biomechanical, and biochemical theories. The vascular theory of glaucoma considers GON as a consequence of insufficient blood supply due to increased IOP and/or other risk factors reducing ocular blood flow [7]. The mechanical theory suggests that GON may result from increased IOP leading to regions of high shear stress and strain in the lamina cribrosa [7]. Over the past few years, there has been mounting evidence in the literature on the possible role of biochemical mechanisms leading to glaucomatous neurodegeneration [8]. These biochemical mechanisms include the role of excitatory amino acids, caspases, protein kinases, oxygen free radicals, nitric oxide, tumor necrosis factor-alpha, neurotrophins, and metalloproteins [8].

An intriguing finding of several studies is that intracranial pressure (ICP) is lower in patients with POAG and NTG [10–12], and a growing body of evidence indicates that glaucoma is a condition that develops from a mismatch in pressures across the lamina cribrosa. The optic nerve, a white matter tract of the central nervous system (CNS), is ensheathed in all three meningeal layers and surrounded by cerebrospinal fluid (CSF) in the subarachnoid space (SAS) with a pressure equivalent to ICP [13]. The movement of CSF along the outside of the optic nerve is well known. When tracers are injected into the cisterna magna or lateral ventricles, they are detected in/around the optic nerve [14, 15]. Thus, in addition to IOP, the optic nerve is exposed to the ICP [6]. The lamina cribrosa separates these two pressurized regions [6]. It forms a pressure barrier between the high-pressure compartment of the intraocular space and the low-pressure compartment of the retrobulbar CSF space [16]. The forces experienced at the level of the optic nerve head are influenced by both IOP and ICP. The difference between the posteriorly directed IOP and anteriorly directed ICP across the lamina cribrosa is known as the trans-lamina cribrosa pressure difference (TLCPD) [6]. The pressure drop that occurs across the lamina cribrosa (IOP-ICP) increases with elevation of IOP or reduction of ICP [6].

Recent insights into CSF biology have revealed the importance of the so-called “glymphatic system” in the clearance of potentially neurotoxic waste products, including amyloid-*β* (A*β*), from the brain via paravascular spaces surrounding cerebral blood vessels [17]. Interestingly, new research now lends support to the hypothesis that a similar system is present in the eye and optic nerve [9]. The discovery of such an “ocular glymphatic system” may be of particular importance for the understanding of the pathophysiology of POAG, given that studies in glaucomatous animal models have shown that A*β* is a likely mediator of pressure-induced RGC death [18]. As discussed in the present paper, an intriguing possibility is that the glymphatic hypothesis of glaucoma may integrate many aspects of the above-noted vascular, biomechanical, and biochemical theories of the disease.

## 2. Discussion

### 2.1. The Brain and the Eye May Have a Similar Glymphatic Clearance Pathway

A novel hypothesis of glaucoma recently proposed by our group is that the disease may result from a dysfunction of the so-called “glymphatic system” [13]. The glymphatic system was first described by Iliff et al. [17] in 2012. The authors defined for the first time a brain-wide network of paravascular pathways in mice, along which a large proportion of subarachnoid CSF circulates through the brain parenchyma, facilitating the clearance of interstitial solutes, including A*β*, from the brain [17]. CSF enters the brain along para-arterial channels for exchange with interstitial fluid (ISF), which is in turn cleared from the brain along paravenous pathways for ultimate clearance via cervical lymphatic vessels [17, 19]. From the SAS, CSF is driven into the Virchow-Robin spaces by a combination of arterial pulsatility, respiration, slow vasomotion, and CSF pressure gradients [19, 20]. The subsequent transport of CSF into the dense and complex brain parenchyma is facilitated by aquaporin-4 (AQP4) water channels which are expressed in a highly polarized manner in astrocytic end-feet ensheathing the cerebral vasculature [19]. AQP4 is essential for water movement across astrocyte cell membranes. A recent study on meningothelial cells that cover the SAS of the optic nerve (including the trabeculae and septa) also demonstrated the presence of AQP4 in human optic nerve sections [21]. Besides removal of metabolic waste products, the glymphatic system may also function to help distribute non-waste compounds, such, such as glucose, lipids, amino acids, and neurotransmitters related to volume transmission, in the brain [19]. Recent analysis shows that the glymphatic system is highly active during sleep and is largely disengaged during wakefulness [19]. It should be noted that while the glymphatic concept assumes transport from the SAS into the parenchyma along periarterial pathways, other studies suggest that the periarterial flow provides a drainage out of the parenchyma [22, 23]. Moreover, the possibility has been raised that the paravascular CSF fluxes observed in previous studies [17, 20] could represent artefacts of changes in ICP resulting from CSF tracer infusion [24]. Obviously, further studies are needed to substantiate the functional significance of the glymphatic concept. Also, studies in other species are warranted. Elucidation of the potential role of the glymphatic system in the human brain is extremely challenging since dysfunction of this system may be an important contributing factor in neurodegenerative diseases such as Alzheimer's disease (AD).

Intriguingly, recent reports presented at the ARVO 2016 Annual Meeting together with preliminary data from our own postmortem study [9] suggest that a similar paravascular clearance system is present in the human optic nerve and retina. In a postmortem study to investigate the possibility of a paravascular fluid circulation, or at least paravascular spaces, in the human optic nerve, we examined cross-sections of human optic nerves by light microscopy after administering India ink by bolus injection into the SAS of the optic nerve (work in progress). The results demonstrated accumulation of India ink in paravascular spaces around the central retinal artery and vein, whereas the lumens of these vessels remained unlabelled [9]. The deposits were located between collagen fiber bundles lining a slit-like space [9]. In addition, in their report presented at the ARVO 2016 Annual Meeting, Hu and colleagues [25] provided evidence for a glymphatic system in human, non-human primate, rat, and mouse retina. Retinas were examined using multimarker immunohistochemistry. An AQP4^+^ glial network ensheathed the entire retinal vascular system, including between blood vessels, and the authors concluded that this may be the anatomical correlate of a retinal glymphatic system. In yet another report presented at the ARVO 2016 Annual Meeting, Löffler and colleagues [26] provided support for lymphatic structures in AD mice retinas similar to the glymphatic system in the brain. The authors investigated possible clearance pathways for A*β* in an AD mouse model (SwAPP/Psen1d9). AD mice retinas exhibited enhanced amyloid precursor protein (APP) production with increased amyloid processing and A*β* accumulation versus wild-type mice. Retinal A*β* plaques were much smaller than in brain. A*β* plaques were located around and in retinal blood vessels. Podoplanin (lymphatic vessel marker) colocalized with A*β* and was increased in AD retinas versus wild-type mice, indicating lymphatic-like vessels in the retina. The authors concluded that A*β* clearance from the retina may occur via lymphatic structures analogous to the described glymphatic system of the brain. These structures appear enhanced in AD.

Intriguingly, in 2015, two independent studies by Aspelund et al. [27] and Louveau et al. [28] reported the presence of dura-associated lymphatic vessels in the brain. These two studies further suggested a connection between the newly identified meningeal lymphatic vessels and the recently discovered glymphatic system. Interestingly, lymphatics in the dura mater of the human optic nerve have previously been described by Gausas et al. [29] and Killer et al. [30]. These findings together with our postmortem observations [9] suggest at least the possibility that a connection may exist between the paravascular fluid circulation and the meningeal lymphatic system in the optic nerve, such as that very recently described between the glymphatic system and the dura-associated lymphatic vessels in the brain. In an editorial discussing our recent publication [9], An et al. [31] concluded by saying that “the linkage between these putative glymphatic systems and the now recognized true lymphatic vessels seen at the termination of the optic nerve SAS around the optic disc is yet to be clarified and is almost certainly going to provide a source for interesting and useful research in the future.”

### 2.2. Glaucoma Considered as an Imbalance between Production and Clearance of Neurotoxins, Including Amyloid-*β*

Considerable evidence indicates that A*β* may be implicated in the development of axonal damage and RGC apoptosis in glaucoma [18, 32–34], suggesting a possible link with AD. Previous findings showed that there is IOP-sensitive increase in A*β* in glaucoma [18, 32–34]. McKinnon et al. [32] reported that rat RGCs subjected to chronic elevation of IOP exhibit caspase-3-mediated abnormal processing of APP with increased expression of A*β*. This suggested a new hypothesis for RGC death in glaucoma involving chronic A*β* neurotoxicity, mimicking AD at the molecular level [33]. Activation of caspases and abnormal APP processing, which includes production of A*β*, are also important events in AD [32]. Guo et al. [18] provided further evidence that A*β* is a likely mediator of pressure-induced RGC death. In a rat model mimicking chronic ocular hypertension, the authors found that A*β* colocalized with apoptotic RGCs [18]. They also demonstrated* in vivo* that A*β* induced significant RGC apoptosis [18]. The authors further provided evidence that targeting A*β* and blocking its effects with combination therapy may represent an effective treatment strategy in glaucoma [18]. By manipulating the A*β* pathway, the authors investigated three different approaches to targeting A*β* in experimental glaucoma and their combination effects: (i) reduction of A*β* formation by a *β*-secretase inhibitor; (ii) clearance of A*β* deposition by an anti-A*β* antibody; and (iii) inhibition of A*β* aggregation and neurotoxic effects with Congo red [18]. The authors showed that combined treatment (triple therapy) was more effective than either single- or dual-agent therapy [18]. Recently, in a study using monkeys with experimental glaucoma, Ito et al. [34] found time-dependent expressions and localization of A*β* in the retina as well as in the optic nerve head after chronic IOP elevation.

The retina is an extension of the CNS, sharing embryological, anatomical, and physiological similarities to the brain [35], and therefore, it seems likely, as suggested in the above-mentioned ARVO reports, that the branches of the central retinal vessels in the retina are also surrounded by paravascular spaces with the same properties as the paravascular spaces in the brain. Furthermore, given that A*β* has been reported to increase by chronic elevation of IOP in glaucomatous animal models and to cause RGC death [18, 32–34], the above-noted findings in the eye presented at ARVO raise the possibility that the clearance of IOP-induced A*β* from the retina may occur via glymphatic structures analogous to the described glymphatic system of the brain and that glaucoma, just like AD, may occur when there is an imbalance between production and clearance of neurotoxins, including A*β* [13, 36].

### 2.3. The Lamina Cribrosa as a Potential Choke Point for Glymphatic Flow between the Optic Nerve and Retina

**Figure 1:**
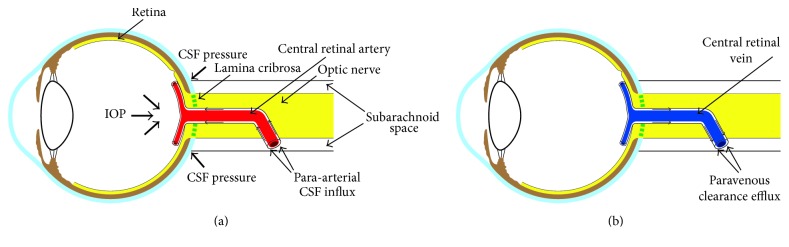
Schematic depiction of our hypothesis. Cerebrospinal fluid enters the paravascular spaces in the retina along a para-arterial influx route around the central retinal artery (a), followed by a paravenous clearance efflux route around the central retinal vein (b) (Figures 1(a) and 1(b) reproduced from [9]).

It should be stressed that the few research data currently available, although encouraging, cannot be considered as proof that a “glymphatic system” exists in the eye and that much more studies are needed to validate this possibility. If evidence further confirms the existence of an “ocular glymphatic system,” it would be interesting to further investigate whether a “paravascular communication” exists between the surroundings of the retinal vascular system and the surroundings of the central retinal vessels in the optic nerve. Such a paravascular “retino-orbital” continuity has previously been suggested [37] and would include a para-arterial CSF influx route around the central retinal artery to enter the paravascular spaces of the retina, followed by a paravenous clearance efflux route around the central retinal vein (Figures 1(a) and 1(b)) [9]. From this point of view, the lamina cribrosa might play a critical role in the paravascular flow between the optic nerve and retina that can cause blockage of this flow with decreased elimination of neurotoxic substances, such as A*β*, and subsequent GON. Histological studies in humans and animals have shown that eyes with glaucoma or elevated IOP often have deformities of the lamina cribrosa such as posterior laminar displacement, laminar thinning, pore deformities, and focal laminar defects [38]. In glaucomatous eyes, alterations in the lamina cribrosa structure may be sufficient to mechanically interfere with glymphatic flow through it. Furthermore, as discussed below, paravascular flow through the lamina cribrosa may be restricted in proportion to the amount of the trans-lamina cribrosa pressure gradient (IOP-ICP/thickness of the lamina cribrosa), and also vascular factors may disturb the physiologic glymphatic flow through it.

### 2.4. Vascular Pulsatility and Cerebrospinal Fluid Pressure as Influencing Factors for Ocular Glymphatic Clearance

Elucidation of a glymphatic clearance pathway in the eye could provide a new unifying hypothesis of glaucoma that can incorporate many aspects of the vascular, biomechanical, and biochemical theories of the disease. Indeed, vascular and mechanical factors may lead to changes in paravascular transport at the site of the lamina cribrosa, influencing glymphatic clearance of toxic substances from the retina. As noted above, cerebral arterial pulsation is a key driving force for glymphatic flow [20]. Analogous to the vascular pulsations in the brain, central retinal artery pulsation could be a key driver of para-arterial CSF influx into the retina. In the eye, high pulsatility efficiency of the central retinal artery may be of paramount importance because the para-arterial CSF influx from the optic nerve to the retina is supposed to occur against the trans-lamina cribrosa pressure gradient. Normally, IOP is higher than ICP [6]. An increase in IOP, a decrease in ICP, or a decrease in the thickness of the lamina cribrosa may increase the pressure barrier against which paravascular flow from the optic nerve to the retina needs to occur. Patients with low ICP and/or high trans-lamina cribrosa pressure barriers and/or central retinal artery pulsatility inefficiency may therefore be more likely to develop glymphatic stasis at the site of the lamina cribrosa, leading to reduced neurotoxin clearance and subsequent GON. Another potentially important aspect may be the dynamics of the pressure changes. A previous study by Morgan et al. [39] investigated the timing of retinal venous pulsation in relation to IOP and ICP pulses. The authors demonstrated a difference in the phasing of the IOP curve and the phasing of the ICP curve with respect to the cardiac cycle, with the ICP curve reaching its height earlier than the IOP curve [39, 40]. In full agreement with these findings, Jonas et al. [40] wondered whether these physiological short-term changes in the TLCPD, potentially even resulting in short-term reversals of the TLCPD, may physiologically be needed to allow the retrograde axoplasmic flow entering the eye. Similarly, we believe that this swinging of TLCPD may also be important for paravascular CSF influx from the optic nerve to the retina.

Interestingly, as mentioned above, recent research suggests that, along with IOP, alterations in ICP may be involved in glaucoma. A growing body of evidence indicates that ICP is lower in patients with POAG and NTG [10–12], and a low ICP gains interest as a new risk factor for glaucoma. This is in line with the present hypothesis. Indeed, if the ICP is too low, fluid flow from the paravascular spaces in the optic nerve to the paravascular spaces in the retina may decline or stop, given that this paravascular flow must cross the trans-lamina cribrosa pressure barrier. It is interesting to note that ICP was found to be lower in NTG patients than in high-tension glaucoma patients [11, 12]. In high-tension glaucoma, IOP-induced generation of toxins might predominate and even mild impairment of glymphatic pathway function might result in glaucomatous optic nerve damage. In NTG, reduced clearance of toxic substances might predominate as a result of glymphatic stasis. Importantly, a previous study in an experimental animal model provided evidence for a possible toxic effect of stagnant CSF on the optic nerve [41]. It was postulated that an accumulation of biologically highly active substances such as lipocalin-like prostaglandin D synthase (L-PGDS), a protein present in the CSF, could exercise a harmful effect on axons and mitochondria of the optic nerve [41]. The highest concentration of mitochondria is located right behind the lamina cribrosa in nonmyelinated axons [41]. The main function of mitochondria is the production of adenosine triphosphate, which is essential for cell survival [42]. Given that the unmyelinated optic nerve has a high relative demand for mitochondrial enzyme activity, the immediate retrobulbar portion of the optic nerve may be particularly vulnerable to toxic effects [41]. Given that astrocytes play a critical role in maintaining the integrity of axon function in the central nervous system and specifically in the optic nerve, Xin et al. [43] investigated the biochemical effects of L-PGDS on the proliferation of astrocytes and on the production of adenosine triphosphate by astrocyte mitochondria in an in vitro model. The authors demonstrated an inhibitory effect of L-PGDS on both proliferation of astrocytes and production of astrocyte adenosine triphosphate [43]. Obviously, L-PGDS is only one of many CSF components with biological activity and other substances could also be harmful.

It is also interesting to note that systemic arterial stiffness, which can occur as a consequence of arteriosclerosis, has been reported to be associated with POAG and NTG [44, 45]. Mroczkowska et al. [44] found systemic arterial stiffness assessed by pulse wave analysis to be comparably increased in early-stage POAG and NTG patients compared with controls. Shim et al. [45] investigated the role of systemic arterial stiffness in glaucoma patients with diabetes mellitus. Their study showed that high brachial-ankle pulse wave velocity (baPWV) was an independent risk factor for glaucoma in diabetes mellitus patients [45]. Mean baPWV of the NTG group was about 7.3% faster than that of the control group [45]. However, mean baPWV of the POAG group was about only 1.3% faster than that of the control group [45]. These results suggested that arterial stiffness is more associated with NTG than with POAG [45]. If the underlying pathophysiology of glaucoma is, at least partly, paravascular transport blockage within the lamina cribrosa, it seems reasonable to expect that systemic arterial stiffness may be a risk factor for glaucoma since arterial hardening may also affect the central retinal artery, resulting in impairment of the arterial pulsation-driven “perivascular pump” in the eye.

The present hypothesis also fits with data on the association between POAG and blood pressure. Pache and Flammer reported hypotension, and in particular a nocturnal drop in blood pressure, as an important risk factor for OAG [46]. Furthermore, the Baltimore Eye Study showed an age-related association between blood pressure and POAG [47]. In particular, systemic hypertension showed a protective effect against glaucoma in younger patients, while it increased the risk of glaucoma in older patients [47]. These age-related findings could be explained by the assumption that the optic nerve potentially benefits from the high perfusion pressure accompanying relatively normal vessels early in life, while chronic vascular changes that limit flow become the dominant influence in older people with narrowed vessel lumen [48]. However, these findings are also consistent with the present glymphatic hypothesis of glaucoma. Indeed, cerebrovascular pulsatility is dependent, at least in part, on systemic blood pressure. Younger people with no blood vessel damage yet may take advantage of high blood pressure by increasing central retinal artery pulsatility, facilitating the paravascular movement of CSF into the retina. However, chronically elevated blood pressure may result in arteriosclerosis and as the blood vessels become rigid with age, there will be reduced central retinal artery pulsatility and subsequent blockage of paravascular flow from the optic nerve to the retina. In the case of nocturnal hypotension, decreased central retinal artery pulsatility may lead to restriction of normal glymphatic flow at the level of the lamina cribrosa during sleep, when glymphatic clearance processes are maximal [19]. Therefore, nocturnal hypotension may have a magnified negative effect on ocular glymphatic clearance compared to hypotension during wakefulness.

Supportive evidence for the role of blood pressure in paravascular flow comes from studies evaluating the role of arterial pulsation in CSF-ISF exchange. The movement of fluid in the perivascular spaces is caused by arterial pulsation resulting from normal heart action [49]. Hadaczek et al. [49] tested the hypothesis that the natural heartbeat could contribute to the distribution and transport of intracranially infused molecules within those spaces. The authors investigated the movement of interstitially infused macromolecules within the CNS in anesthetized rats with either high blood pressure and heart rate (induced by epinephrine) or low blood pressure and heart rate (induced by blood withdrawal) and in rats euthanized just before the infusion (no heart action) [49]. The rats with high blood pressure and heart rate displayed a significantly larger distribution of the infused molecules within the injected site and more extensive transport of those molecules [49]. Their results confirmed a rapid spread of molecules that cannot be explained by diffusion as the sole mechanism [49]. As heart action contributed substantially to broad distribution of the molecules, the authors proposed that the pulse acts as a pump to distribute particles infused into the interstitium of the brain along the conduit of the perivascular space to sites deeper in the parenchyma and remote in the brain [49]. In a more recent study, Iliff et al. [20] used* in vivo* two-photon microscopy in mice to visualize cerebral arterial wall pulsatility within surrounding paravascular spaces. Systemic administration of the adrenergic agonist dobutamine increased blood pressure and heart rate [20]. A significant elevation in pulsatility was observed along penetrating arteries [20].* In vivo* and* ex vivo* analysis of fluorescent CSF tracer influx into and through the brain parenchyma demonstrated that increasing pulsatility with dobutamine accelerated the rate of paravascular CSF influx into brain tissue [20]. It is important to note that arterial undulation depends on the expansion and contraction of the arterial wall with each pulse, which depends not only on how much the heart contracts, but also on the resistance of the circuit defined by diameter and elasticity of the blood vessel [49]. With the onset of arteriosclerosis, the artery walls become more rigid, the amplitude of pulsations is reduced, and the passage of fluid along the blood vessel walls is impaired [49]. Thus, regardless of blood pressure, there may, under this circumstance, be no fluid flow outside the blood vessel [49]. The findings from the above-mentioned animal studies are completely in line with the reported association between blood pressure and POAG and therefore, we speculate that the relationship between blood pressure and paravascular flow may be of importance when evaluating the association between blood pressure and glaucomatous damage. We hypothesize that restriction of normal glymphatic flow at the level of the lamina cribrosa may be a new potential mechanism promoting the development of glaucoma in patients with nocturnal hypotension and older patients with systemic hypertension.

## 3. Conclusions

The pathophysiology of POAG is still largely unknown, although a joint contribution of vascular, biomechanical, and biochemical factors is widely acknowledged, thus making POAG rather a syndrome than a disease. Since glaucoma is a leading cause of blindness in the world, exploring its underlying pathophysiological mechanisms is extremely important and challenging. Evidence from recent studies appears supportive of the hypothesis that a “glymphatic system” exists in the eye and optic nerve, analogous to the described “glymphatic system” in the brain. As discussed in the present paper, elucidation of a glymphatic clearance pathway in the eye could provide a new unifying hypothesis of glaucoma that can incorporate many aspects of the vascular, biomechanical, and biochemical theories of the disease. We are aware that the results from only a few studies until now do not scientifically prove the existence of an “ocular glymphatic system.” Much more study in the fields of eye and glymphatic research is needed to validate this possibility. Even though nothing conclusive can yet be said, these first reports suggesting a paravascular transport system in the eye and optic nerve are encouraging and, if confirmed, may offer new perspectives for the development of novel diagnostic and therapeutic strategies for this devastating disorder. We therefore wish to encourage future research in this area.
